# Arteriovenous fistula as a complication of transradial coronary angiography: a case report

**DOI:** 10.1186/1752-1947-7-21

**Published:** 2013-01-14

**Authors:** Payam Dehghani, Jennifer Culig, Darshan Patel, Greg Kraushaar, Paul Schulte

**Affiliations:** 1 Prairie Vascular Research Network; Department of Cardiology, University of Saskatchewan, 2574 Linner Way, Regina, SK S4V 1K3, Canada; 2 Prairie Vascular Research Network; University of Saskatchewan, Regina, SK, Canada

## Abstract

**Introduction:**

Iatrogenic arteriovenous fistula is a vascular condition that may result from coronary angiography. Many case reports have described arteriovenous fistula occurrence after coronary angiography using the transfemoral access route, but rarely as a complication of using the transradial approach. We report a rare case of a patient with arteriovenous fistula following transradial artery coronary angiography.

**Case presentation:**

A 62-year-old Caucasian man underwent emergent coronary angiography using the right radial artery approach. One month after angiography, he discovered a turbulent sound near the access site. A right radial arteriovenous fistula was found upon duplex ultrasound investigation. The patient was treated conservatively. At 1-year follow-up, the arteriovenous fistula was unchanged and the patient remained hemodynamically stable and asymptomatic.

**Conclusion:**

Iatrogenic arteriovenous fistula is a rare vascular complication of transradial artery coronary angiography. The natural history of arteriovenous fistula is benign and is thought to resolve spontaneously; therefore, a conservative approach, as opposed to surgical ligation, is recommended as the first-line treatment.

## Introduction

Two to five percent of patients presenting with an acute coronary syndrome (ACS) experience major bleeding [1,2], a clinical factor which has been established as an independent predictor of mortality [3,4]. As a substantial portion of bleeding occurs at the femoral site during transfemoral coronary angiography and/or angioplasty, the field of interventional cardiology has turned its attention to accessing the heart from the radial artery. This is a superficial and readily compressible site that, in comparison to the transfemoral approach, has been proven to reduce major bleeding and is associated with reduction in mortality [5]. In addition, the transradial artery (TRA) approach is associated with increased comfort and decreased time to ambulation.

Vascular complications such as access site hematomas, retroperitoneal hematomas, pseudoaneurysms, arteriovenous fistula (AVF) and arterial dissection are related more to femoral artery puncture sites than to radial access sites. Although reported with a very rare incidence in the medical literature [6], there are no publications describing non-surgical, conservative treatment of AVF from the TRA approach. We report the first case of AVF that was managed conservatively using this technique and review the literature regarding the pathophysiology, risk factors for, natural history of and potential management strategies for AVF in the radial artery.

## Case presentation

**Figure 1:**
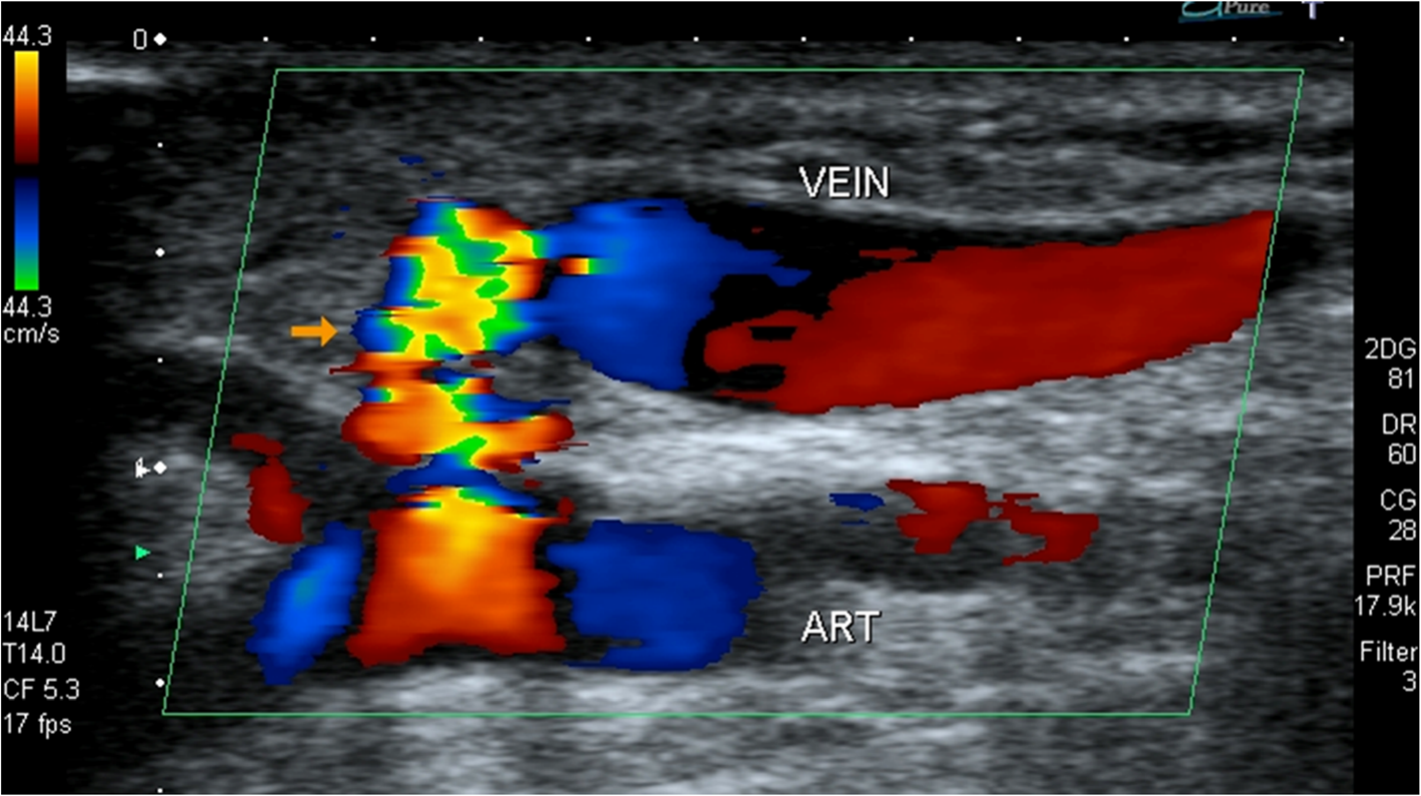
An ultrasound scan of the right radial artery revealed the arteriovenous fistula seen as turbulent high velocity flow (orange arrow) between the vein and the radial artery (ART).

A 62-year-old Caucasian man with a body mass index (BMI) of 26kg/m^2^ and no classic risk factors for coronary artery disease presented with an ACS requiring emergent coronary angiography. After confirming dual arterial supply of the palmar arch with a normal Allen’s test, angioplasty of the proximal and mid-left anterior descending artery was carried out using a 6-French, 23cm radial sheath (Cordis Corporation, Miami, FL, USA) in the right radial artery. Bivalirudin, with an initial bolus of 0.75mg/kg followed by 1.75mg/kg/hour for the duration of the procedure, was used for anticoagulation. The radial artery sheath was removed immediately after completion of percutaneous coronary intervention (PCI), and hemostasis was achieved by application of an adjustable plastic clamp on the radial artery. The clamp was gradually released over 2 hours while the access site was monitored for bleeding or hematoma, then it was removed after satisfactory access site hemostasis was achieved. After an uneventful hospital stay, the patient was discharged on the appropriate medical therapy. One month later he complained of a “swishing” sensation in his right forearm. He did not complain of finger claudication or numbness. His physical examination confirmed a palpable thrill at the right radial puncture site. An ultrasound (Figure 1) with color Doppler imaging showed an AVF with turbulent, high-velocity flow at the site of communication. There was no circulatory compromise to the hand, and the Allen’s test remained normal.

The patient was sent to a vascular surgeon, who elected to follow the patient conservatively. A repeat ultrasound performed 1 year later showed exactly the same anatomy, without any increase in the fistulous connection. At this time, the patient continues to have no discomfort at the access site.

## Discussion

In comparison with transfemoral access, TRA coronary angiography has repeatedly been shown to improve patient satisfaction [7,8], reduce resource use [8-10] and lower risk of vascular complications [5,7]. In a recent prospective registry of 455 patients undergoing TRA coronary angiography, of whom only 15% underwent an intervention, the most common access site complications were radial artery occlusions in 25%, pseudoaneurysms in 0.6% and AVFs in 0.9%, and no major bleeding was reported [11]. Although most of the medical community has come to interpret this registry’s unacceptably high rates of radial artery occlusion with caution [12], the remainder of the findings are consistent with other published findings of low rates of vascular compromise [5,7]. No specific mention was made of treatment strategies for patients with AVF in the registry cohort. Even more relevant to our case is the largest randomized trial to compare radial and femoral access, the RIVAL study [7], which included 7021 patients with ACS undergoing intervention. Although the primary outcome, a composite of death, myocardial infarction, stroke or non–coronary artery bypass graft–related bleeding at 30 days, was not statistically significant between the radial and femoral approaches, major vascular complications, defined as large hematoma, pseudoaneurysm, AVF and ischemic limb needing surgery, occurred in 1.4% of radial arms compared to 3.8% of femoral arms (*P* < 0.001).

Acquired AVF caused by transfemoral coronary angiography is not uncommon, owing to the frequency of the groin as the site of percutaneous arterial and venous access. However, AVF in the arm is very rare and has been reported very infrequently [13-15]. In the RIVAL study, 5 (0.14%) of the 3514 patients undergoing transfemoral PCI had AVF, whereas none of the 3507 patients in the TRA arm were reported to have AVF. Regardless of whether the upper or lower extremity is involved, the pathophysiology of iatrogenic AVF is the same. During access, needle deviation through a venous tributary can lead to an unnoticed combined artery and vein puncture. Most of the time the communication between the artery and vein will seal spontaneously. However, when the communication does not seal, an AVF may form.

The technique and experience of the operator, compression at the access site, management of hemostasis after catheterization, closure devices used and nursing care must be considered when evaluating the patient’s risk of developing vascular complications post-angiography. Potential ways to minimize the incidence of AVF include (1) operator experience, (2) limiting the number of times the same artery is accessed, (3) using a sheath size that is less than the arterial diameter and (4) using ultrasound-guided needle placement to provide anatomic information such as diameter, tortuosity and proximity of the radial artery to the vein. Reviewing the literature on the incidence and risk factors of AVF caused by femoral angiography can be instructive as well. In the largest series of its kind, Kelm *et al*. found that significant predictors of AVF in this patient population were high heparin dosage, coumadin therapy, puncture of the left groin, arterial hypertension and female gender [16]. Interestingly, other than anticoagulation with bivalirudin, our patient had none of these risk factors and his artery was accessed after the first puncture.

What is the natural history of persistent AVF? If the AVF is large enough, two potential hemodynamic consequences may be seen. First, significant hemodynamic shift can occur from the higher systemic vascular resistance in the artery to the lower resistance in the venous circuit. The ensuing increase in stroke volume from increased venous return may potentiate high output failure. Second, AVF in the lower extremity, in the setting of pre-existing vascular disease, can lead to the potential worsening of ischemic symptoms. The experience derived from treating AVF due to femoral artery catheterization suggests that most cases can resolve spontaneously. In Kelm *et al*.’s description of the cohort of patients with transfemoral AVF, one-third of the AVFs closed spontaneously within 1 year [16]. In another report, 81% of patients’ AVFs resolved spontaneously within 200 days after femoral angiography [17]. Looking at the natural history of iatrogenic AVF in high-risk hemodialysis patients can also be instructive. Keeping in mind the different patient population as well as the larger diameter of the brachial artery, the most recent report, which described a cohort of 628 hemodialysis patients with surgically caused AVFs with 8 years of follow-up, 16% experienced one or more complications [18]. Hemorrhage, failure to mature and aneurysms were the most common complications seen in the early, immediate and late groups, respectively [18]. Limb ischemia and cardiac insufficiency, the two complications with the most dreaded hemodynamic sequelae, were seen in only 3.6% of the entire cohort.

There are four potential therapeutic approaches for patients with persistent iatrogenic AVF. First, surgical repair, the approach taken for all the reported cases of radial AVF undergoing coronary angiography [13-15], has been advocated as the intervention of choice. This is an invasive option with its own reported rates of peri-operative morbidity and mortality [17]. Surgical options depend on the size and location of the AVF and include partial resection, ligation, excision and repair. Second, implantation of a covered stent, previously described in the femoral artery [19-21], has inherent limitations in the radial artery, given the small size of the artery, the potential likelihood of restenosis and the unknown natural history. Third, ultrasound-guided compression, which has been well-described in the population with AVF after use of the trans-femoral approach, may be an option. There have been no reports of ultrasound-guided compressions in the radial artery, however, and the experience of femoral complications suggests low rates of success [22,23]. Fourth, conservative therapy, the approach taken in our patient, is a viable option, given the benign natural history of AVF and the fact most seal spontaneously. This case challenges the dogma that AVF caused by use of the TRA approach needs to be addressed surgically, as was the case in all the previous publications describing this complication [13-15]. It is our opinion that as long as radial AVF is of no inconvenience to the patient, is not enlarging and does not cause neurovascular compromise and/or cardiac insufficiency, it should be treated conservatively without any intervention.

Although many complications can arise with iatrogenic AVF, there is enough evidence in the literature to suggest that its natural history is benign and therefore can be managed expectantly. Appropriate follow-up of stable patients presenting with AVF post-TRA catheterization includes clinical and ultrasound assessment.

## Conclusion

Iatrogenic AVF is a rare vascular complication of transradial coronary angiography that may be treated conservatively without any interventions.

## Patient’s perspective

I am a 62-year-old male Caucasian with little medical knowledge or background. I am writing as my own perspective on conditions that led to development of a fistula in my right wrist.

In late July 2010, usually while walking, I had been noticing an unusual sensation in my upper arms and across my chest. It was enough of an oddity to motivate me to go to our family doctor. He prescribed a nitroglycerin spray and blood work that I had done at a local lab.

A few days later, prior to having received any lab results, I went to a kids’ camp to work as a volunteer custodian. I couldn’t have been too worried about health risks as I neglected to take the nitroglycerin spray with me to the camp.

By the end of the first day of cutting grass at the camp I felt unusually exhausted. Although I had no pain, my daughter (a registered nurse), convinced me to go to the nearby hospital at Fort Qu’Appelle to get checked out. Blood samples were taken and indicated high enzyme levels. The hospital staff advised me that I had had a heart attack and I was sent by ambulance to the Regina General Hospital.

The following day, my cardiologist carried out an angioplasty procedure and inserted two stents. A few days later, I was released from hospital. However, I soon noticed what I called “my vibrating wrist” in my right wrist, in the area that the angioplasty catheter had been inserted. I showed this to Dr. Dehghani who said that I had a fistula, an unexpected, abnormal connection between a vein and artery.

I was sent for an ultrasound examination and to an appointment with a vascular surgeon. I stated that the fistula was not really bothering me. The surgeon recommended that no further action be taken since risks of the status quo were likely less than if I were to have vascular surgery to try to correct the fistula.

Over the past 18 months since the angioplasty procedure, my “vibrating wrist” has become gradually less noticeable. The fistula has never bothered me and now, I have mostly forgotten that it is still there.

## Consent

Written informed consent was obtained from the patient for publication of this case report and accompanying images. A copy of the written consent is available for review by the Editor-in-Chief of this journal.

## Competing interests

The authors declare that they have no competing interests.

## Authors’ contributions

PD is senior author of the paper. PD is the cardiologist who took care of the patient and was the primary writer of the manuscript. JC conducted an extensive literature search, spoke with the patient and contributed significantly to the intellectual content of the report. DP contributed significantly to the discussion section of the preliminary draft of the manuscript. GK and PS interpreted the radiographic images and made intellectual contributions to the report. All authors read and approved the final manuscript.
